# Prevention of Mother-to-Child Transmission (PMTCT) of HIV services in Adama town, Ethiopia: clients’ satisfaction and challenges experienced by service providers

**DOI:** 10.1186/1471-2393-14-57

**Published:** 2014-02-01

**Authors:** Anteneh Asefa, Getnet Mitike

**Affiliations:** 1 School of Public and Environmental Health, Hawassa University, Hawassa, Ethiopia; 2 School of Public Health, Addis Ababa University, Addis Ababa, Ethiopia

## Abstract

**Background:**

The coverage and uptake of prevention of mother-to-child transmission (PMTCT) of HIV services has remained very low in Ethiopia. One of the pillars of improving quality of health services is measuring and addressing client satisfaction. In Ethiopia, information about the quality of PMTCT services regarding client satisfaction is meager.

**Methods:**

A facility-based cross-sectional study using quantitative methods was conducted in Adama town. We interviewed 423 pregnant women and 31 health providers from eight health facilities. Satisfaction of clients was measured using a standard questionnaire adapted from the UNAIDS best practices collection on HIV/AIDS. Bivariate and multivariate logistic regression analyses were used to identify factors associated with clients’ satisfaction.

**Results:**

About three-fourth (74.7%) of clients reported that they were satisfied with the PMTCT services provided by the health facilities. However, a much lower proportion (39%) of the total respondents (pregnant women who underwent an ANC follow-up session), said they received and understood the messages related to mother-to-child transmission (MTCT) of HIV and PMTCT. The main challenges reported by service providers were lack of training, lack of feedback on job performance and inadequate pay. Clients’ satisfaction with PMTCT service was found to be associated with liking the discussion they had with their counselor, non-preference to a different counselor with regards to sex and/or age and not seeing the same ANC counselor before and after HIV test.

**Conclusion:**

Although 74.7% of clients were satisfied, the majority did not have a good understanding of the counseling on MTCT and PMTCT. We recommend more efforts to be exerted on improving provider-client communication, devising ways of increasing clients’ satisfaction and designing an effective motivation strategy for service providers to enhance the status of PMTCT services.

## Background

In the year 2011, around 330,000 children acquired HIV infection. This represents a 43% decline from 560,000 children reported to have been infected with HIV in 2003. More than 90% of new HIV infection amongst children in the year occurred in Sub-Saharan Africa, home to 92% of pregnant women living with HIV. Furthermore, only 59% of pregnant women living with HIV in Sub-Saharan Africa received antiretroviral therapy or prophylaxis in the year 2011 [1]. In Ethiopia, one of the countries severely hit by the epidemic, the national HIV prevalence estimate was at 2.1% in 2010/2011. In the same year, there were around 137,494 new HIV infections, 10% of which occurred amongst children. Moreover, only 9.3% of the estimated numbers of HIV positive pregnant women were provided with antiretrovirals (ARVs) for PMTCT in 2011 [2,3].

In Ethiopia, the proportion of HIV positive mothers identified at PMTCT sites has increased from 1.88% in 2006 to 22.1% in 2010 and during the same period the number of health facilities providing PMTCT services has shown an 8-fold increment from 171 (21.3%) to 1,352 (61.9%) [4]. Among pregnant women who have received antenatal care from those PMTCT service sites, only 75.5% of them were tested for HIV [2].

Developing and implementing a successful PMTCT program require a comprehensive approach including ensuring availability of minimum number of staff, providing training and cascading a continuum of care for mothers, infants and their families after delivery [5]. Ethiopia adopted its first PMTCT guideline in 2001. Then the second and the third (currently used) guidelines were formulated in 2007 and 2011 respectively [6,7]. All guidelines follow the four pronged approach to PMTCT which is recommended by the World Health Organization [8,9]. The Federal Ministry of Health of Ethiopia, recognizing the challenge, has come up with an accelerated plan to guide program implementation and coordination for the coming one year. This emergency approach is intended to rapidly increase service provision sites, improve quality of services and increase utilization so as to reach the ambitious goals set in the health sector development program of providing ARVs for PMTCT to 77% of eligible pregnant women by 2015 [3,10].

According to the Ethiopian Demographic and Health Survey of 2011, only 9.9% of mothers deliver at health institutions and only 34% of mothers receive antenatal care from health professionals [11]. Strengthening the integration of PMTCT services within maternal, newborn and child health (MNCH), sexual and reproductive health, and family planning services in health facilities is one of the critical priorities outlined for reaching the PMTCT targets [3].

One of the pillars of improving quality of health services is measuring and addressing client satisfaction. Thus, this study was conducted to understand the level of satisfaction of clients with PMTCT services and the challenges which providers face in the implementation of PMTCT services in Adama town, Ethiopia. The findings of the study are expected to inform policy makers and providers in their efforts to accelerate the uptake and coverage of PMTCT services through addressing gaps related to clients’ satisfaction, providers’ perception and services provision.

## Methods

### Study design

A facility-based cross-sectional study which encompassed quantitative methods (client exit interview, and providers’ survey) was done in Adama town from March to April 2010.

### Study setting

The study was conducted in Adama town which is the capital of East Shoa zone and which is found 100 kms south of the nation’s capital, Addis Ababa. The study was conducted at a government run hospital, three government run health centers, two private hospitals and two private health centers.

### Study population

The study population included selected women who visited the above health institutions for antenatal care services and all PMTCT service providers in those health institutions.

### Sample size and sampling

The sample size for clients’ exit interview was determined using a single population proportion formula by taking an assumption that 50% of the clients would be satisfied by PMTCT services (to get a conservative estimate of the sample size as there was no information on the proportion of clients satisfied with PMTCT services in the country), with 5% precision, 95% confidence and possible non-response of 10%. Hence, the calculated sample size was 423 mothers for respondents of the survey amongst clients. On the providers side, all health workers (n = 31) who were directly involved with the provision of PMTCT services were included. Allocation of sample to health institutions was made proportionally based on review of the flow of new ANC attendees in each institution in the preceding two weeks of data collection period. Clients were selected by systematic random sampling using clients’ registration books from each facility as a sampling frame.

### Data collection

A standardized questionnaire from the UNAIDS best practice collection which basically focuses on HIV/AIDS counseling service assessment was used to assess client satisfaction [12]. The tool was purposively selected since it is the most commonly used tool in low resource settings to generate useful information. The tool inquires information on: mothers’ obstetric history, reason for seeking ANC, comfortableness with the way counselors handle clients, presence of the same counselor before and after HIV test, privacy during counseling, topics of discussion during HIV counseling, understandability of messages, and happiness with stay during ANC. Furthermore, other variables (socio-demographic variables, waiting time, the sex of counselor and question merely asking level of satisfaction) were merged with it to gain more contextual understanding of clients’ satisfaction. The questionnaire was translated to Amharic and back to English for maintaining consistency. A total of 28 items were included in the exit interview questionnaire.

Health providers were surveyed using a baseline assessment tool for PMTCT service assessment prepared by Family Health International’s Institute for HIV/AIDS [13]. The tool was used to assess the level of contribution and challenge/s faced by service providers in the provision of PMTCT services.

### Data collectors

Exit interviews with clients were administered by trained data collectors who were female high school graduates. The health providers’ survey was conducted by trained clinical nurses.

### Data quality assurance

Pre-testing of the data collection tools was conducted at Bishoftu hospital using 5% of the total sample size to identify any weakness in the organization and structuring of the research instruments. Bishoftu Hospital is situated in a neighboring town where the population shares similar attributes as that of Adama. Following the pre-test, the tools were improved in terms of their clarity, understandability and simplicity in collecting the data required for the study.

### Data analysis and interpretation

Data were first coded then cleaned and entered using EPI INFO software version 3.5.1. Descriptive frequencies and rating of satisfaction across facilities visited by clients were done using SPSS version 16. Bivariate and multivariate analyses were done to identify factors related to satisfaction. Satisfaction level was assessed using a five level Likert scale (1-very dissatisfied, 2-dissatisfied, 3-indifferent, 4-satisfied, and 5-very satisfied). To calculate the proportion of satisfied clients, those who responded to be “very satisfied” and “satisfied” were categorized to be satisfied clients and those who responded to be “indifferent”, “dissatisfied”, and “very dissatisfied” were categorized as unsatisfied.

### Ethical considerations

Ethical clearance was obtained from the Institutional Review Board of Faculty of Medicine, Addis Ababa University. Written permission was obtained from the Oromiya Regional Health Bureau and the institutions in which the study was carried out. Additionally, written consents were obtained from all clients and health providers who participated in the study.

## Results

### Socio-demographic and obstetric characteristics of respondents

A total of 423 ANC attending pregnant women participated in the exit interviews which were carried out to assess the respondents’ degree of satisfaction. The socio-demographic characteristic of the clients is described in Table 1. More than forty one percent (41.4%) were in the age group 21-25 years and the mean age was 24.8 years (SD ± 4.9 years). More than ninety percent (93.9%) of the respondents were married. Regarding educational status of clients, 60 (14.2%) of the respondents are illiterate whereas 164 (38.8%) and 45 (10.6%) of the respondents had a secondary school (grade 9-12) and college or more level of education respectively. Of the total mothers interviewed, the majority 223 (52.7%) of them were in the third trimester of pregnancy while 161 (38.1%) were in the second trimester of pregnancy.

**Table 1:** Socio-demographic characteristics of women who came for ANC services, Adama, March 2010

***Characteristics***	***Options***	***Frequency***	***Percentage***
Age in years	15–20	95	22.5
	21–25	175	41.4
	26–30	108	25.5
	31–40	44	10.4
	41 and above	1	0.2
Place of residence	Adama town	361	85.3
	Out of Adama town	62	14.7
Religion	Orthodox	252	59.6
	Muslim	96	22.7
	Protestant	67	15.8
	Catholic	5	1.2
	Others	3	0.7
Marital status	Married	397	93.9
	Single	16	3.8
	Divorced	6	1.4
	Widowed	2	0.5
	Separated	2	0.5
Maximum educational status	Illiterate	60	14.2
	Illiterate but able to write and read	9	2.1
	Grade 1–4	101	23.9
	Grade 5–8	44	10.4
	Grade 9–12	164	38.8
	College and above	45	10.6
Ethnic origin	Oromo	158	37.4
	Amhara	141	33.3
	Tigre	28	6.6
	Gurage	75	17.7
	Others	21	5.0
Occupational status	House wife	268	63.4
	Merchant	40	9.5
	Government employee	29	6.9
	Student	23	5.4
	Daily laborer	30	7.1
	Others	33	7.8

### Counseling services received by respondents on the date of visit

On the day of the interview, clients who had undergone counseling were asked about issues related to HIV that were discussed with the antenatal care counselors. The result showed that discussions focused on issues related to undergoing an HIV test, issues related to receiving the results and issues arising from HIV tests taken previously, as confirmed by 209 (49.4%), 160 (37.8%), and 186 (44.0%) of the clients respectively (Table 2).

**Table 2:** Pregnant women’s reason for coming to the ANC clinics, topics about HIV discussed with ANC attendants, waiting time and pregnant women’s number of ANC visits, Adama, March 2010

***Characteristics***	***Frequency***	***Percentage***
***Reason for coming to the ANC center***		
For antenatal care only	366	86.5
To test for HIV	6	1.4
To receive treatment to protect her baby from HIV	4	1.0
For antenatal care and to test for HIV	45	10.6
For antenatal care, to test for HIV, To receive treatment to protect her baby from HIV	2	0.5
Topics discussed with mothers about HIV*		
Having an HIV test	209	49.4
Receiving test results	160	37.8
Issues arising from an HIV test taken Some time ago	186	44.0
MTCT and PMTCT	220	52.0
***Understandability of messages conveyed on MTCT/PMTCT (n = 220)***		
Claimed to have understood the messages	165	75.0
Claimed not to have understood the messages	55	25.0
***Trimester of pregnancy***		
First	39	9.2
Second	161	38.1
Third	223	52.7
Waiting time before meeting ANC counselor		
< 15 minutes	188	44.4
≥ 15 minutes	235	55.6

*multiple response.

More than half, 220 (52.0%) of the clients were counseled on MTCT and PMTCT while only 10 (2.4%) of them were counseled on infant feeding. Regarding understandability of the message conveyed during clients’ counseling sessions, among clients who received counseling on MTCT/PMTCT (n = 220), 165 (75.0%) claimed to have understood the message. However, from the total respondents who underwent an ANC follow-up session with health providers (n = 423), only 165 (39.0%) received and claimed to have understood the message on MTCT/PMTCT (Table 2).

**Figure 1:**
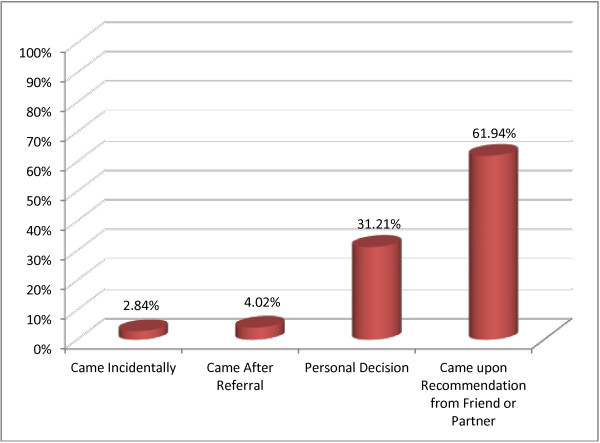
How the pregnant women came to visit the health facility where they had antenatal care for the first time, Adama, March 2010 (n = 423).

Concerning clients’ reasons for coming to ANC service delivery sites, ANC follow-up was mentioned as a primary reason by 86.5% of respondents. Clients were asked about how they first came to the health institution they visited and more than 61.0% of them reported that their visit was upon recommendation from their friends or partners. Only 4.0% of clients came due to referral from other health institutions (Figure 1).

### Waiting time experienced by pregnant women

The mean waiting time for clients to get to the counseling service was 24.5 minutes and the range is calculated to be 359 minutes. When the waiting time was stratified by type of health institution, the waiting time was 21.5 minutes and 41.5 minutes in governmental and in private health institutions respectively. The average duration of stay of clients with their health care provider in the clinics was 12.8 minutes.

### Pregnant women’s satisfaction with PMTCT services during ANC

Of the total respondents, 96.0% reported that they were happy with the session they had during the date of interview. On the other hand, the percentages of women who felt comfortable with their counselor’s handling of clients and who perceived presence of enough privacy were 93.4% and 73.8% respectively (Table 3).

**Table 3:** Subjective response of clients towards satisfaction based questions, Adama, March 2010

**Subjective questions**	**Response**		**Total, N (%)**
	**Yes, n (%)**	**No, n (%)**	
Are you happy with the session you had today?	406 (96.0)	17 (4.0)	423 (100)
Did you feel comfortable with your counselors handling of the client?	395 (93.4)	28 (6.6)	423 (100)
Was there enough privacy during your counseling?	312 (73.8)	111 (26.2)	423 (100)
Do you wish you had a different counselor (different sex, older, younger)?	76 (18.0)	347 (82.0)	423 (100)
Were you able to see the same counselor for discussion both before and after the test?	145 (34.3)	278 (65.7)	423 (100)
Is there anything you did not like during the discussion about HIV/AIDS?	28 (6.6)	395 (93.4)	423 (100)
Would you have preferred that HIV/AIDS not be discussed during your antenatal visit?	20 (4.7)	403 (95.3)	423 (100)

**Figure 2:**
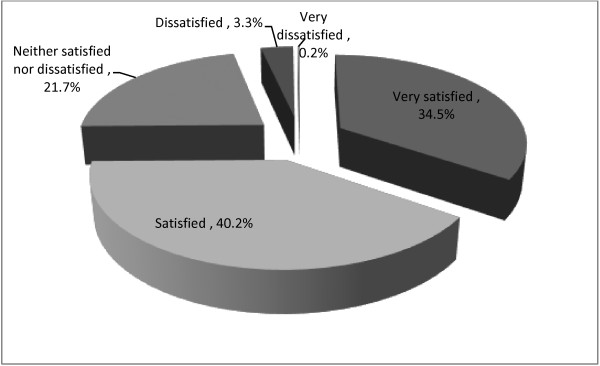
Level of satisfaction of ANC visitors with PMTCT services at the service delivery facilities, Adama, March 2010 (n = 423).

The percentage of clients who were satisfied with PMTCT services during their stay at the service delivery facility on the day of their ANC visit was 74.7% (71.2% in public health institutions and 87.8% in private health institutions) (Figure 2). This proportion includes all those who reported to be very satisfied (34.5%) and satisfied (40.2%). Multivariate analysis (logistic regression) controlled for age, education, address, marital status and occupation was carried out to identify variables associated with clients’ satisfaction. Pregnant women’s satisfaction with PMTCT services was associated with liking the discussion they had with their counselor [AOR = 0.13, 95% CI: 0.04, 0.41], non-preference to a different counselor with regards to sex and/or age [AOR = 0.04, 95% CI: 0.19, 0.82] and not seeing the same ANC counselor before and after HIV test [AOR = 0.26, 95% CI: 0.12, 0.49] (Table 4).

**Table 4:** Logistic regression analysis of satisfaction by selected explanatory variables, Adama, March 2010

**Explanatory characteristics**	**Satisfied**	**Unsatisfied**	***COR (95% CI)***	***AOR (95% CI)***
**Is there anything you did not like during the discussion about HIV/AIDS?**				
Yes	17	11	1.00*	
No	299	96	0.47 (0.26, 1.10)	0.13 (0.04, 0.41)**
**Do you wish you had a different counselor (different sex, older, younger)?**				
Yes	21	55	1.00*	
No	295	52	0.07 (0.04, 0.12)**	0.04 (0.19, 0.82)**
**Were you able to see the same ANC counselor for discussion both before and after HIV test?**				
Yes	88	57	1.00*	
No	228	50	0.34 (0.26, 0.53)**	0.26 (0.12, 0.49)**
**Waiting time before meeting the ANC counselor**				
≤ 15 minutes	147	41	1.00*	
> 15 minutes	169	66	1.40 (0.89, 2.19)	1.19 (0.66, 2.14)
**Recommended to come to the health institution by others**				
Yes	209	53	1.00*	
No	107	54	1.99 (1.26, 3.11)**	1.87 (1.00, 3.51)

*Reference group **statistically significant association; Controlled for age, education, residence, marital status and occupation.

### Work related characteristics reported by service providers

All health professionals included in the study (n = 31) work in more than one department and 14 (45.2%) of them spend most of their time in ANC unit. Among the total providers, 21 (67.7%) were involved in the provision of HIV counseling, 28 (90.3%) in infant feeding counseling and 29 (93.5%) in obstetrics care. The average hours spent per day by providers in counseling MTCT was 5.2 hours and 22 (71.0%) of the providers said that their workload has increased since the introduction of the PMTCT services.

### Challenges perceived by service providers in the provision of PMTCT services

All health providers included in the study (31) reported that there were no incentives for providing PMTCT services. Among health professionals who provide obstetrical care (n = 29), twenty one (72.4%) of them were trained in safe obstetric practices in caring for HIV-positive women. During the survey, providers were given a set of possible challenges they may encounter in providing PMTCT services and the top three most difficult problems reported to be encountered in performing their job in providing PMTCT related services were lack of feedback on job performance, inadequate pay and lack of training, which were reported by 93.5%, 77.4% and 58.1% of service providers respectively.

## Discussion

It is known that counseling on MTCT and PMTCT is the most important service out of the services that make up the package of PMTCT services to be offered to all pregnant women visiting ANC clinics. This counseling was offered to 220 (52.0%) pregnant women who were interviewed on the day of their ANC visit. In contrast to our finding, MTCT and PMTCT were discussed on the day of visit for 74.7% of the pregnant mothers interviewed in Addis Ababa [14]. Among those who have received counseling on MTCT and PMTCT (n = 220) in our study, 165 (75%) claimed to have understood the message conveyed. However, in the study conducted in Addis Ababa, they only assessed whether the information was received and did not inquire whether clients felt that they understood the messages.

Clients’ average waiting time and average duration of stay with their health care provider were 24.5 minutes and 12.8 minutes respectively. This finding was by far better than the report from Kenya. In a PMTCT program in Kenya more than 90.0% of the clients waited for an average of 150 minutes in addition to the amount of time set by the program guideline for obtaining the services, which is 90 minutes [15].

In this study, there was a preference to be counseled by the same counselor in pre and post test counseling during the ANC visit. These findings might be related to clients’ concerns regarding issues of confidentiality. In regards to HIV testing and counseling for PMTCT, it is recommended that the person offering pre-test information provides the post test counseling [16,17]. However, this may be a challenge in many settings of developing countries because of shortage of trained human resource [18].

In this study the level of satisfaction was not affected by either age or sex of the counselor. This may indicate that satisfaction might be more affected by other factors than the socio-demographic characteristics of counselors. In this study, a high proportion (74.7%) of clients reported that they were satisfied with the service they had received. In this respect, there was no statistically significant difference in satisfaction among clients who experienced waiting time of less than or equal to 15 minutes, and those who had to wait more. However, long waiting time (more than 15 minutes), poor counseling service, and lack of privacy were reported to be the main challenges in the provision of maternal, neonatal, child & adolescent health services in Ethiopia in the five year period, 2005 to 2010 [10].

This high proportion of satisfied clients might be due to the fact that clients may not report dissatisfaction with services even when services seem to be poor. A research carried out in a university hospital in Brazil also showed that patients generally stated to be satisfied with the rendered service and positively appraised the quality of provided service, that is, even when critical factor that undermined quality of service provision seem to exist [19]. This may over time result in normalization of egregious violations of standards of practice in health care delivery systems [20]. Besides, normalization of disrespect and abuse by individuals and community is identified to be one among the contributors to disrespect and abuse exercised by service providers during maternity health services [21].

In this study, clients who liked the discussion they had with their counselor were less likely to be satisfied with the PMTCT service they received. This surprising finding may be due to the fact that the situations faced by clients at health institutions may not be relevant to them if compared to the quality of attention received which can largely affect their satisfaction [19].

In our study, out of the total PMTCT service providers interviewed, 22 (71.0%) of them said that their workload had increased since the introduction of the PMTCT service and all of them did not get any incentives for providing those PMTCT services. A consultation report from Kenya also validates the fact that the introduction of HIV/AIDS prevention and care into the MCH setting has meant that health workers have been asked to greatly expand their responsibilities and tasks [18]. Others have reported that the additional responsibilities are rarely accompanied by financial and other types of compensation or that they lead to hasty services that affect the quality of the counseling services [22,23].

This study has deployed a multidimensional approach to understand clients’ satisfaction with PMTCT service and challenges experienced by service providers in providing PMTCT service in the study area; however, the scope is limited in addressing all factors which may affect clients’ satisfaction and which may pose challenges on service providers. In addition, the study is also limited as it does not look into rural-urban differences.

## Conclusions

This study has shown that clients’ satisfaction with PMTCT service was sub-optimal and PMTCT service providers are confronting challenges which can hinder them from providing quality services. Hence, key actors and implementers of PMTCT program need to address bottlenecks which hamper delivery of full package of PMTCT services in line with the national PMTCT guideline. Based on the findings of this study, offering quality counseling on MTCT and PMTCT to all pregnant women, strengthening providers’ capacity and motivation technique to help them deliver quality, comprehensive PMTCT interventions are recommended. Furthermore, improving provider-client communication and devising ways of increasing clients’ satisfaction with PMTCT services is advised.

## Competing interests

The authors declare that they have no competing interests.

## Authors’ contributions

Both authors contributed equally to this work.

## Pre-publication history

The pre-publication history for this paper can be accessed here:

http://www.biomedcentral.com/1471-2393/14/57/prepub
